# Effects of foliar boron application on seed composition, cell wall boron, and seed δ^15^N and δ^13^C isotopes in water-stressed soybean plants

**DOI:** 10.3389/fpls.2013.00270

**Published:** 2013-07-23

**Authors:** Nacer Bellaloui, Yanbo Hu, Alemu Mengistu, My A. Kassem, Craig A. Abel

**Affiliations:** ^1^Crop Genetics Research Unit, Plant Physiology, USDA-ARSStoneville, MS, USA; ^2^College of Life Science, Northeast Forestry UniversityHarbin, China; ^3^Crop Genetics Research Unit, USDA-ARSJackson, TN, USA; ^4^Plant Genomics and Biotechnology Laboratory, Department of Biological Sciences, Fayetteville State UniversityFayetteville, NC, USA; ^5^Corn Insects and Crop Genetics Research, USDA-ARS, Iowa State UniversityAmes, IA, USA

**Keywords:** boron, carbon, cell boron, fatty acids, nitrogen, oil, protein

## Abstract

Limited information is available on the effects of foliar boron (B) application on soybean seed composition. The objective of this research was to investigate the effects of foliar B on seed composition (protein, oil, fatty acids, and sugars). Our hypothesis was that since B is involved in nitrogen and carbon metabolism, it may impact seed composition. A repeated greenhouse experiment was conducted where half of the soybean plants was exposed to water stress (WS) and the other half was well-watered. Foliar boron (FB) in the form of boric acid was applied twice at a rate of 1.1 kg ha^−1^. The first application was during flowering stage, and the second application was during seed-fill stage. Treatments were water stressed plants with no FB (WS–B); water stressed plants with FB (WS+B); watered plants without FB (W–B), and watered plants with FB (W+B). The treatment W–B was used as a control. Comparing with WS–B plants, B concentration was the highest in leaves and seed of W+B plants (84% increase in leaves and 73% in seed). Seeds of W+B plants had higher protein (11% increase), oleic acid (27% increase), sucrose (up to 40% increase), glucose, and fructose comparing with W–B. However, seed stachyose concentrations increased by 43% in WS–B plants seed compared with W–B plants. Cell wall (structural) B concentration in leaves was higher in all plants under water stress, especially in WS–B plants where the percentage of cell wall B reached up to 90%. Water stress changed seed δ^15^N and δ^13^C values in both B applied and non-B applied plants, indicating possible effects on nitrogen and carbon metabolism. This research demonstrated that FB increased B accumulation in leaves and seed, and altered seed composition of well-watered and water stressed plants, indicating a possible involvement of B in seed protein, and oleic and linolenic fatty acids. Further research is needed to explain mechanisms of B involvement in seed protein and fatty acids.

## Introduction

Soybean is a major crop in the world. Soybean seed has nutritional value because of its content of protein, oil, sugars, isoflavones, and minerals. Soybean seed contains five main fatty acids [two saturated: palmitic (C16:0) and stearic (C18:0) acids; three unsaturated: oleic (C18:1), linolenic (C18:2), and linolenic (C18:3) acids]. Protein concentration in soybean ranges from 341 to 568 g kg^−1^ of total seed weight, with a mean of 421 g kg^−1^, and oil ranges from 83 to 279 g kg^−1^, with a mean of 195 g kg^−1^ (Wilson, 2004). Palmitic acid ranges from 10 to 12%, stearic from 2.2 to 7.2% (Cherry et al., 1985), oleic acid (24%), linoleic acid (54%), and linolenic acid (8%) (Schnebly and Fehr, 1993). Seed contains about 9–12% total soluble carbohydrates, including sucrose (4–5%), raffinose (2%), and stachyose (3.5–4.5%) (Wilson, 1995; Wilson et al., 1995). Seed macro- and micro-nutrients concentrations for soybean were previously reported (Zobiole et al., 2010; Bellaloui et al., 2011), and their ranges were depended on genotype, maturity, environmental factors including temperature, drought, and disease pressure (Bellaloui et al., 2011, 2012a,b). Because soybean seed contains phenolics, including lignin and isoflavones which are antioxidants, it was thought to have health benefits against osteoporosis, cancer, and heart disease for humans (Messina et al., 1994; Potter et al., 1998; Sakthivelu et al., 2008), and a role in plant disease resistance and defense mechanisms (Paxton, 1980; Graham, 1983; Graham and Webb, 1991; Dixon, 1999). Seed with higher oleic acid and lower linolenic acid are desirable for oil stability, long-term shelf storage, and processing. Higher linoleic and linolenic acids are undesirable for soybean processing because of their oxidative instability and the needs for their hydrogenation, leading to transisomers, which are associated with increased incidence of heart disease (Rakow and McGregor, 1973). In contrast, monounsaturated fatty acids such as oleic acid are less susceptible to oxidative changes during refining, storage, and frying.

Boron is an essential nutrient for plant growth, development, and quality (Pilbeam and Kirkby, 1983; Marschner, 1995; Brown et al., 1999; Dordas, 2006; Dordas et al., 2007). Boron has mainly a structural role (Hu and Brown, 1994; Brown et al., 2002), although metabolic roles of B were also reported. For example, the role of B in nitrogen fixation (Bolaños et al., 1996), nodules (Bolaños et al., 1994; Carpena et al., 2000), and nodullin protein (ENOD2) in nodule parenchyma cells and malfunction of oxygen diffusion barrier (Bonilla et al., 1997) was reported. So far, there is no convincing evidence that B directly affects nitrogen metabolism (Shelp, 1993; Marschner, 1995; Bonilla et al., 1997). The role of B in carbohydrates metabolism (Marschner, 1995), especially sugar alcohols (Bellaloui et al., 1999; Brown et al., 1999), phenolic metabolism (Marschner, 1995), ion uptake (Goldbach, 1985; Marschner, 1995), plasma membrane-bount H+ ATPase (Schon and Blevins, 1990; Camacho-Cristóbal and González-Fontes, 2007; Camacho-Cristóbal et al., 2008), and cell wall structure and membrane integrity (Schon and Blevins, 1990; Marschner, 1995) was previously reported.

Although the literature on the structural and metabolic role of B (Pilbeam and Kirkby, 1983; Marschner, 1995; Brown et al., 2002) is well-established, the results obtained from research on the effects of B on soybean seed yield (Reinbott and Blevins, 1995; Ross et al., 2006) are still inconsistent, and information about foliar B effects on composition (protein, oil, fatty acids, sugars) is almost non-existent. For example, foliar B fertilizer at 0, 0.28, 0.56, 1.12, and 2.24 kg B ha^−1^ did not affect soybean yield at one site, but increased seed yields at three sites (Reinbott and Blevins, 1995; Ross et al., 2006). Ross et al. (2006) reported that B application at V2 (vegetative stage) increased yields by 13% compared with the application at R2 (flowering stage). It was concluded that the application of 0.28–1.12 kg B ha^−1^ during early vegetative or reproductive stages was sufficient to produce near maximal yields (Ross et al., 2006). Schon and Blevins (1990) found that foliar split application of B, twice during flowering, increased the number of pods/branch, and a total of 0.56 kg ha^−1^ was optimal for pods/branch increase. The source of the inconsistency of the effect of B on soybean could be due to genotype, environmental factors such as heat and drought, and their interactions. Therefore, the objective of this research was to investigate the effect of foliar B on soybean seed protein, oil, fatty acids (palmitic, stearic, oleic, linoleic, and linolenic acids), and sugars. Our hypothesis was that since B is involved, directly or indirectly, in nitrogen and carbon metabolism, it may impact seed protein, oil, and sugars accumulation.

## Materials and methods

### Growth conditions

A repeated greenhouse experiment was conducted at the Delta States Research Center, Stoneville MS. Soybean cultivar Hutcheson seed were germinated in flat trays in vermiculite. Uniform size seedlings at about V1 stage were transplanted into 9.45 L size pots filled with field soil. Soil used was a Dundee silt loam (fine-silty, mixed, active, thermic Typic Endoqualfs) with pH 6.3, 1.1% organic matter, a cation exchange capacity of 15 cmol/kg, and soil textural fractions of 26% sand, 56% silt, and 18% clay, and average B concentration of 0.72 mg·kg^−1^. The soil contained an abundant native population of *B. japonicum*. Water stress was imposed as follows: soil in pots were weighed, saturated with deionized water, and then left to drain, and then weighed again to obtain the water field capacity using soil water sensors inserted in pots and read by daily by Soil Moisture Meter (WaterMark Company, Inc., Wisconsin, USA). Water stressed plants were kept between −90 and −100 kPa, and watered plants were kept between −15 and −20 kPa (this was considered field capacity for the control plants) (Bellaloui et al., 2010a). Soybean plants were divided so that half of the soybean plants had been exposed to water stress (WS) (soil water potential between −90 and −100 kPa) and the other half had been well-watered (soil water potential between −15 and −20 kPa). Foliar boron (as boric acid) was applied twice at a rate of 1.1 kg ha^−1^. The first application was conducted during flowering stage (R1-R2), and the second application was conducted during seed-fill stage (R5-R6) using hand sprayer, and measures were taken to avoid boron drift to the control plants. Tween 20 at 0.05% was used as surfactant in foliar B application. Dripping of B solution was avoided during the application. For the control plants (W–B and WS–B), only deionized water and Tween 20 at 0.05% were foliar applied (Schon and Blevins, 1990). Leaf samples for B measurements were taken 5 days after the first application at flowering stage. Nitrogen fixation measurements were conducted on plants at R1–R2 stages. Mature seed at R8 (harvest maturity) were collected for seed composition analyses and weighed. Plants were considered fully matured when they reached R8 according to Fehr and Caviness (1977). Four replicates were used in each treatment and in each experiment. Each pot had three individual plants. Greenhouse conditions were about 34 ± 9°C during the day and about 28 ± 7°C at night with a photosynthetic photon flux density (PPFD) of about 800–2300 μmol·m^−2^ ·s^−1^, as measured by Quantum Meter (Spectrum Technology, Inc., Illinois, USA). The range of light intensity reflects a bright, sunny, or cloudy day. The lighting in the greenhouse was a mixture of natural light, bulb light (60 W), and cool white (250 W). The two experiments were conducted simultaneously during the normal growing season (from April to September) to simulate the growing season of Early Soybean Production System used in the midsouth USA.

### Boron determination

Boron concentrations were determined in mature seeds at harvest (R8) and fully expanded leaves at flowering stage (R1-R2) in all replicates across all B and water treatments using the Azomethine-H method (Lohse, 1982; Dordas, 2006; Dordas et al., 2007). Briefly, a 1.0-g seed sample was ashed at 500°C and then extracted with 20 ml of 2 M HCl at 90°C for 10 min and filtered. The filtered mixture was transferred to plastic vials, and a 2-ml sample of the solution was added to 4 ml of buffer solution (containing 25% ammonium acetate, 1.5% EDTA, and 12.5% acetic acid) and 4 ml of freshly prepared azomethine-H solution (0.45% azomethine-H and 1% of ascorbic acid) (John et al., 1975). Boron concentration in leaves and seeds was determined after color development, and samples were read at 420 nm using a Beckman Coulter DU 800 spectrophotometer (Fullerton, CA). Boron concentration in soil was determined at the Soil, Plant, and Water Laboratory, University of Georgia, Athens, GA using inductively coupled plasma spectrometry (ICP) using Thermo Elemental, Thermo Jarrell-Ash model 61E ICP, USA.

### Cell wall boron determination

Cell wall B in leaves was determined based on the method of Hu and Brown (1994). Briefly, samples of the fully expanded leaves were homogenized with an ice cold mortar and pestle in cold water. Then, the homogenate was centrifuged at 1000 g for 10 min. The residue was washed three times with 10 ml of 80% ethanol and once with 10 ml of methanol:chloroform mixture (1:1, v/v). The precipitate was then washed with 10 ml of acetone. The samples were dried and ashed for cell wall B determination as described above.

### Seed analysis for protein, oil, and fatty acids

Mature seeds were analyzed for protein, oil, and fatty acids. About 25 g of seed from each plot was ground using a Laboratory Mill 3600 (Perten, Springfield, IL). Analyses were conducted by near infrared reflectance (Wilcox and Shibles, 2001; Bellaloui et al., 2009b) using a diode array feed analyzer AD 7200 (Perten, Springfield, IL). Calibrations were developed by the University of Minnesota, using Perten's Thermo Galactic Grams PLS IQ software. The calibration curve was established according to AOAC methods (1990a,b). Analyses of protein and oil were performed based on a seed dry matter basis (Wilcox and Shibles, 2001; Boydak et al., 2002), and fatty acids were analyzed on an oil basis.

### Seed analysis for sucrose, raffinose, and stachyose

Mature seed at R8 were analyzed for sucrose, raffinose, and stachyose concentrations. About 25 g of seed from each plot were ground using a Laboratory Mill 3600 (Perten, Springfield, IL). Analyses were conducted by near infrared reflectance (NIR) (Wilcox and Shibles, 2001; Bellaloui et al., 2010b) using an AD 7200 array feed analyzer (Perten, Springfield, IL). Analyses of sugars were performed based on a seed dry matter basis (Wilcox and Shibles, 2001; Boydak et al., 2002).

### Seed glucose determination

Glucose was determined following an enzymatic reaction using Glucose (HK) Assay Kit from Sigma, USA, Product Code GAHK-20. In this reaction, glucose is phosphorylated by adenosine triphosphate (ATP) in a reaction catalyzed by hexokinase. The resulted glucose-6-phosphate (G6P) is then oxidized to 6-phosphogluconate in the presence of oxidized nicotinamide adenine dinucleotide (NAD) in a reaction catalyzed by glucose-6-phosphate dehydrogenase (G6PDH). During this oxidation, an equimolar amount of NAD is reduced to NADH, and the consequent increase in absorbance at 340 nm is directly proportional to glucose concentration in the sample. The Glucose (HK) Assay Reagent was reconstituted according to the manufacturers' instructions (Sigma, USA, 2012a,b) in 20 ml deionized water. Mature seed samples were ground using a Laboratory Mill 3600 (Perten, Springfield, IL) to obtain uniform particles. A random ground sample of 0.1 mg was extracted with deionized water. The sample solution was heated by heat plate to aid extraction. The extract was diluted to 1:100 with deionized water to obtain a range of 0.05–5 mg glucose ml^−1^. A sample of 100 μ l was added to 1 ml of the Glucose Assay Reagent in a cuvette and incubated at room temperature for 15 min. A sample blank consisting of 100 μ l of sample and 1 ml of deionized water, and a reagent blank consisting of 1 ml of Glucose Assay Reagent and 100 μ l of deionized water were also prepared. After 15 min, the absorbance was read at 340 nm using a Beckman Coulter DU 800 spectrophotometer (Fullerton, CA). The concentration of glucose was expressed as mg g dwt^−1^.

### Seed fructose determination

Fructose in seed was determined enzymatically according to Fructose Assay Kit from Sigma, USA, Product Code FA-20. In this reaction, fructose is phosphorylated by ATP in a reaction catalyzed by hexokinase. Then, fructose 6-phosphate is converted to G6P by phosphoglucose isomerase (PGI). Then, oxidation of G6P to 6-phosphogluconate takes place in the presence of NAD in the reaction catalyzed by glucose-6-phosphate dehydrogenase (G6PDH). During this oxidation, an equimolar amount of NAD is reduced to NADH, and the consequent increase in absorbance at 340 nm is directly proportional to fructose concentration in a sample. Mature seed samples were ground using a Laboratory Mill 3600 (Perten, Springfield, IL) as described above in the “GLUCOSE DETERMINATION.” A random sample of 0.1 mg was extracted with deionized water. The sample solution was heated by heat plate to aid extraction. A dilution of the extract was diluted to 1:100 with deionized water to obtain a range of 100–1000 μg fructose ml^−1^. A sample of 100 μ l was added to 2 ml of the Glucose Assay Reagent and 0.02 ml PGI in a cuvette and incubated at room temperature for 15 min. A sample blank consisting of 100 μ l of sample and 0.02 ml deionized water was prepared. A sample of Glucose Assay Reagent blank and PGI blank were also prepared as recommended by the manufacturer. After 15 min, samples were read at absorbance 340 nm using a Beckman Coulter DU 800 spectrophotometer (Fullerton, CA). The concentration of fructose was expressed as mg g dwt^−1^.

### Acetylene reduction assay

Nitrogenase activity (NA) was assayed using the acetylene reduction assay as described by Hardy et al. (1968), Zablotowicz et al. (1981), and Bellaloui and Mengistu (2008). Briefly, roots with nodules intact were excised and incubated in 1 L Mason jars. Four roots were placed in the Mason jars and sealed. A 10% volume of air was removed and replaced with an equal volume of acetylene. Duplicate 1.0 ml gas samples were removed after 1 h of incubation at room temperature and analyzed by gas chromatography (An Agilent HP6960, Agilent Technologies, Wilmington, DE) for ethylene formation. The gas chromatograph was equipped with manual injector, injector loop, sample splitter, flame ionization detector (FID), and thermal conductivity detector (TCD). A 0.25 ml sample of gas was directed into a 30 m length × 0.53 mm i.d. alumina megabore column, connected to the FID, and 0.25 ml of sample was injected into a HP- PLOT D column (30 m length × 0.53 mm i.d. megabore with 40 μm film; 1905D-Q04) connected to the TCD using helium as a carrier gas. The integration of chrotographs was conducted using Chem Station software. Ethylene standard curves were performed for each day of analysis. Nodules dry weight was determined by drying the nodules in an oven at 60°C for 4–5 days.

### Analysis of natural abundance δ^15^N and δ^13^C isotopes

Natural abundance of δ^15^N and δ^13^C was determined on about 0.9 mg of ground seeds as previously described (Delwiche and Steyn, 1970; Shearer and Kohl, 1986; Peoples and Herridge, 1990; Bellaloui et al., 2008). Isotopic analysis was conducted using a Thermo FinniGlyn Delta Plus Advantage Mass Spectrometer with a FinniGlyn ConFlo III, and Isomass Elemetal Analyzer (Bremen, Germany). Isodat software version 2.38 was used to calculate Delta values. The elemental combustion system was Costech ECS 4010 with an autosampler (Bremen, Germany).

### Experimental design and statistical analysis

The experimental design was a randomized complete block in a split-plot design with water treatment as main plot and B treatment as sub-plot. Four replicates were used in each treatment, and in each experiment. Analysis of variance was conducted using Proc GLM in SAS (SAS Institute, 2001). Means were separated by Fisher's least significant difference test at 5% probability level.

## Results

Analysis of variance showed that water stress and B treatments had significant effects on seed weight, nitrogen fixation, and seed composition components (Table 1). As expected, experiment had significant effects on some seed components, indicating that the two environments had different conditions due to light and temperature distribution. There were no interactions between water stress, B treatments and experiment, indicating that B treatment had the same effect in each experiment (Table 1). Therefore, data from both experiments were pooled and combined.

**Table 1:** **Analysis of variance of the effects of foliar boron on leaves and seed components**.

**Source of variability**	**Grain weight**	**NF**	**Total B**	**Cell wall B**	**Protein**	**Oil**	**Palmitic**	**Stearic**	**Oleic**	**Linoleic**	**Linolenic**
Experiment (E)	^*^	^**^	NS	NS	^*^	^*^	NS	NS	^**^	NS	NS
Treatment (T)	^***^	^**^	^***^	^***^	^***^	NS	^*^	^***^	^*^	^***^
Water stress (WS)	^***^	^*^	^***^	^**^	^***^	^*^	NS	^*^	^***^	^*^	^***^
E × T	NS	NS	NS	NS	NS	NS	NS	NS	NS	NS	NS
E × WS	^*^	^*^	NS	NS	NS	NS	NS	^*^	NS	NS	NS
T × WS	^*^	^*^	NS	NS	NS	NS	NS	NS	NS	NS	NS
E × T × WS	NS	NS	NS	NS	NS	NS	NS	NS	NS	NS	NS

* *P* ≤ 0.05;

** *P* ≤ 0.01;

*** *p* ≤ 0.001.

### Seed weight, nodule mass, and nitrogen fixation

**Figure 1:**
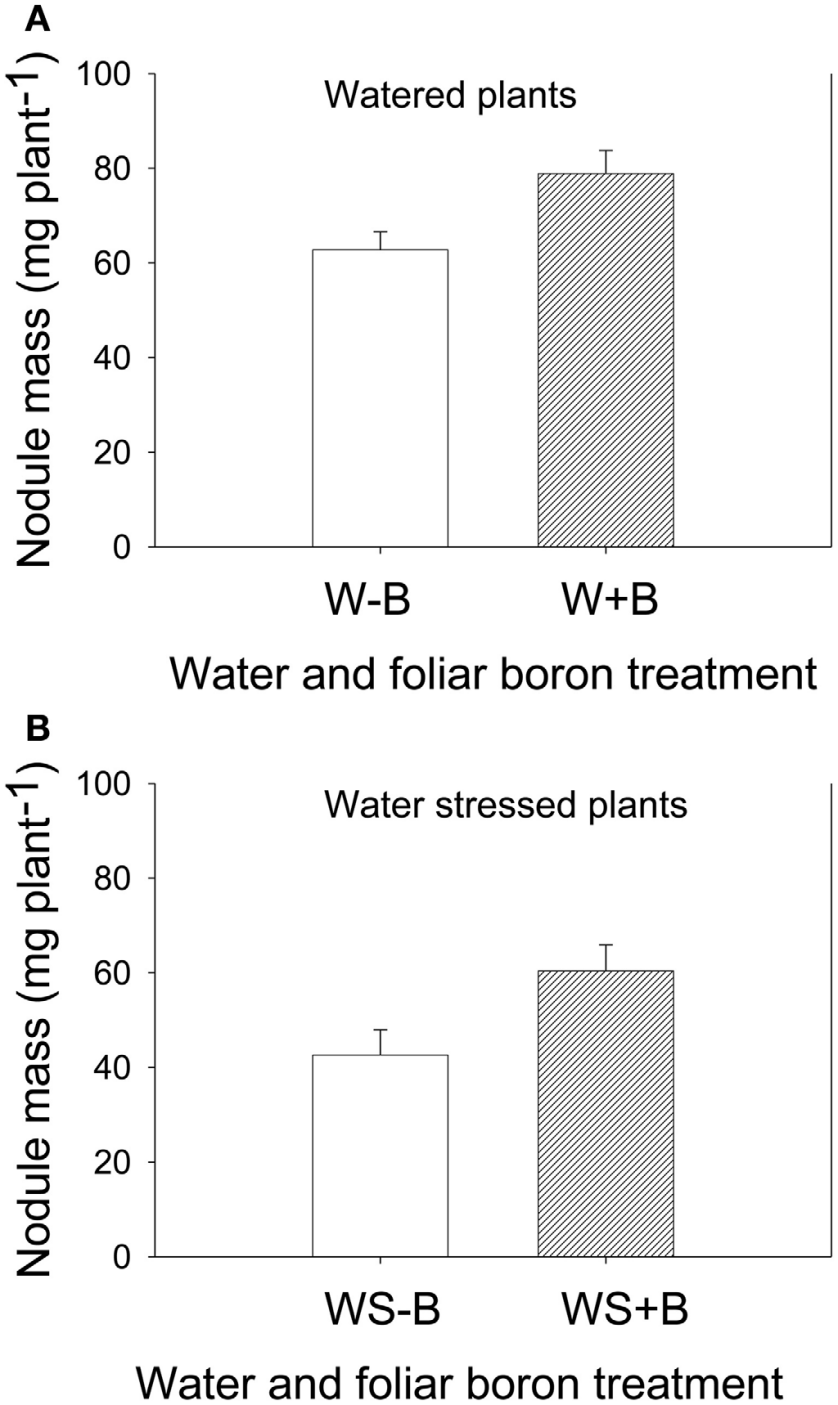
**Effect of foliar boron application (1.1 kg ha^−1^) on nodule mass (g plant^−1^) in watered plants (A) and water stressed soybean plants (B).** Treatments were watered plants without foliar B (W–B), and watered plants with foliar B (W+B), water stressed plants with no foliar B (WS–B); water stressed plants with foliar B (WS+B).

Foliar B application resulted in higher seed weight (weight of 100 seeds and seed weight per plant) for both well-watered and water stressed plants (Table 2). Seed weight in water stressed plants was significantly lower than well-watered plants, indicating an inhibitory effect of water stress. The seed weight was lowest in WS–B plants and highest in W+B plants. Foliar B application had similar trend effects on nodule mass where the highest mass was observed in W+B plants and the lowest was in WS–B plants (Figure 1). Compared with WS–B or WS+B, W–B or W+B plants had higher rate of nitrogen fixation, but W+B plants had higher rate compared with W–B plants (Figures 1A,B).

**Table 2:** **Effects of foliar boron (1.1 kg B ha^−1^) and water stress on seed weights (g), seed protein, oil, and fatty acid concentrations (g constituent kg^−1^ dwt)**.

**Water treatment**	**Boron treatment**	**Weight (100-seed weight)**	**Grain weight Plant^−1^**	**Protein**	**Oil**	**Palmitic (C6:0)**	**Stearic (C18:0)**	**Oleic (C18:1)**	**Linoleic (C18:2)**	**Linolenic (C18:3)**
W	−B	13.2^b^	15.4^b^	395^d^	22.4^a^	10.3^a^	3.2^b^	21.1^c^	55.3^a^	8.6^a^
	+B	16.1^a^	18.5^a^	437^b^	19.5^b^	11.4^a^	4.7^a^	26.7^b^	51.4^b^	6.5^c^
WS	−B	9.30^d^	10.5^d^	427^c^	18.3^d^	11.6^a^	4.4^a^	27.5^d^	57.9^a^	7.6^b^
	+B	11.5^c^	12.6^c^	457^a^	17.1^c^	10.7^a^	3.1^b^	31.4^a^	48.6^c^	6.1^c^

Fatty acids was expressed on oil basis.

Means within a column followed by the same letter are not significantly different at the 5% level as determined by Fishers' LSD test.

W, watered plants; WS, water stressed plants; –B, plants with no foliar boron application; +B, plants with foliar boron application.

### Seed composition

Seed protein was higher in W+B, WS–B, and WS+B than in W–B plants (Table 2). Seed oil had the opposite trend where the highest concentration of oil was in W–B and the lowest was in WS–B plants (Table 2). Saturated fatty acids, palmitic and stearic, were the least responsive to foliar B, although stearic showed inconsistency (Table 2). Foliar B resulted in highest concentration of oleic acid in W+B and WS–B plants. The lowest concentration of oleic acid was observed in W–B plants (Table 2). The highest concentration of oleic acid was accompanied by the lowest concentrations of linoleic and linolenic acids, showing inverse trends.

### Seed sugars

Concentrations of seed sucrose, glucose, and fructose were higher in W–B and W+B plants than in WS–B and WS+B plants, and the concentration of seed sucrose was lowest in WS–B (Table 3). Foliar B application resulted in an increase of seed sucrose concentration in water stressed plants (Table 3). The concentrations of seed raffinose showed inconsistent results. Seed stachyose concentrations showed the opposite trend to sucrose, glucose, and fructose where concentrations of seed stachyose were higher in water stressed plants than in watered plants. The highest stachyose concentration was observed in WS–B plants. Both glucose and fructose responded positively to foliar application of B in watered or water stressed plants (Table 3).

**Table 3:** **Effects of foliar boron (1.1 kg B ha^−1^) and water stress on seed sugar concentrations (mg g^−1^ dwt)**.

**Water treatment**	**Boron treatment**	**Sucrose**	**Raffinose**	**Stachyose**	**Glucose**	**Fructose**
W	−B	51.5^b^	11.2^b^	29.4^c^	2.03^b^	0.71^b^
	+B	72.4^a^	12.1^b^	31.4^c^	2.63^a^	0.93^a^
WS	−B	37.1^d^	15.2^a^	42.1^a^	1.82^d^	0.44^d^
	+B	43.7^c^	12.1^b^	37.5^b^	2.06^c^	0.61^c^

Means within

a column followed by the same letter are not significantly different at the 5% level as determined by Fishers' LSD test.

W, watered plants; WS, water stressed plants; –B, plants with no foliar boron application; +B, plants with foliar boron application.

### Boron concentration in leaves and seed

**Figure 2:**
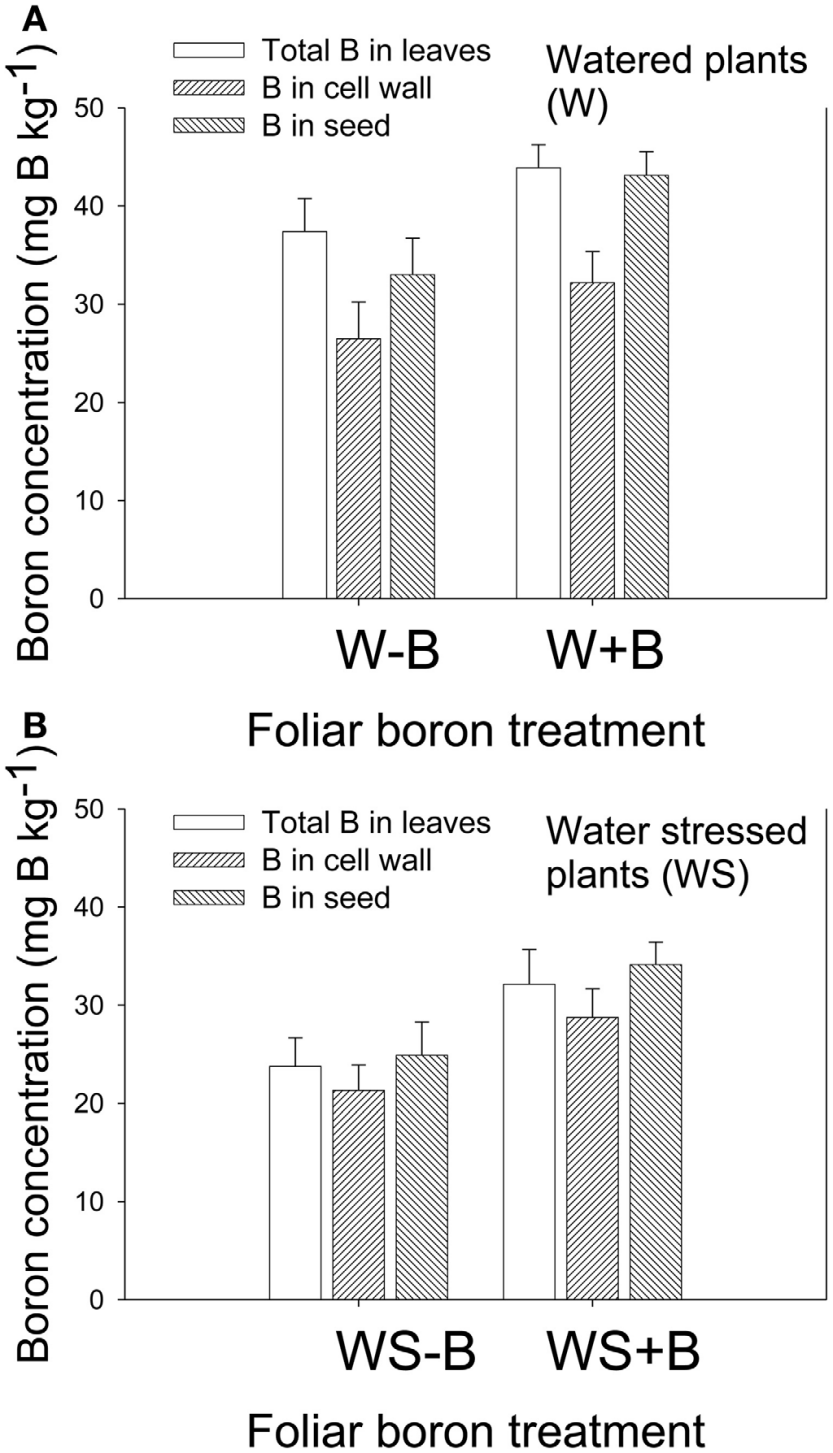
**Effect of foliar boron application (1.1 kg ha^−1^) on total and cell wall boron in leaves, and total boron in seed in watered plants (A) and water stressed plants (B).** Treatments were watered plants without foliar B (W–B), and watered plants with foliar B (W+B), water stressed plants with no foliar B (WS–B); water stressed plants with foliar B (WS+B).

Foliar B application resulted in higher B concentration in leaves and seed in both watered and water stressed plants (Figures 2A,B). Cell wall boron concentration increased with the increase of foliar B, but the percentage contribution of cell wall B to total B decreased. For example, percentage of cell wall B in W–B plants was 80.5% and in W+B plants 73.3%. The contribution of cell wall B to the total B was significantly higher in WS–B and WS+B. For example, the percentage of cell wall B in WS–B was 89.8 and in WS+B was 89.6%. The increase of B in leaves and seed in watered or water stressed plants following foliar B application indicated that soybean plants responded positively to B application.

### Seed natural abundance δ^15^N and δ^13^C isotopes

**Figure 3:**
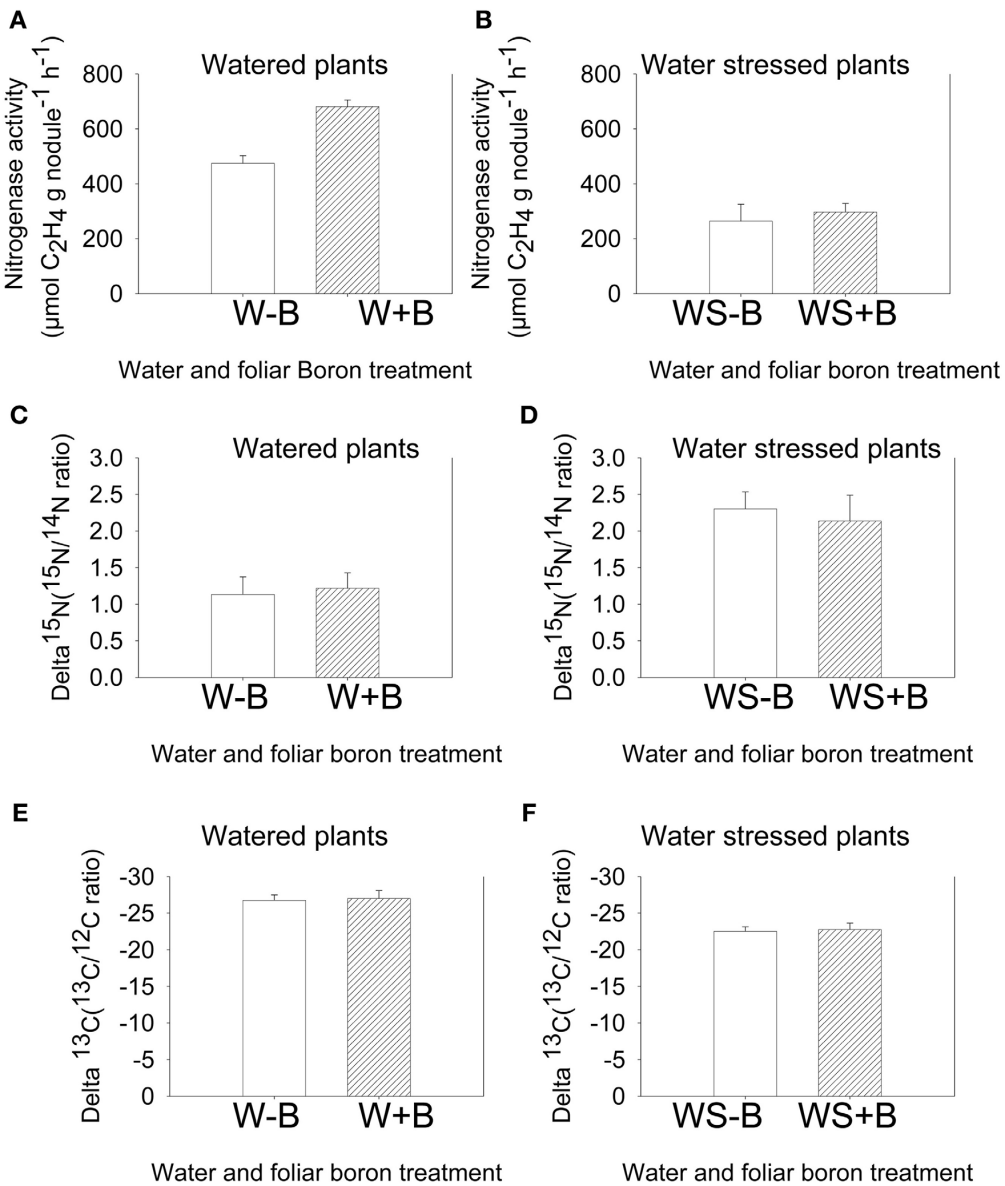
**Effect of foliar boron application (1.1 kg ha^−1^) on nitrogenase activity (nitrogen fixation rate) in watered plants (A) and water stressed plants (B), natural abundance δ^15^N in seed of watered plants (C) and water stressed plants (D), and natural abundance δ^13^C in seed of watered plants (E) and water stressed plants (F).** Treatments were watered plants without foliar B (W–B), and watered plants with foliar B (W+B), water stressed plants with no foliar B (WS–B); water stressed plants with foliar B (WS+B).

The standard nitrogenase fixation assay (acetylene reduction assay) instantly measures the activity of nitrogenase activity at a given time under specific conditions. This allows comparison of treatments measured at the same time, but any extrapolation to the estimate of fixation over a growing season would not be appropriate because environmental conditions, especially temperature, drought, and light intensity may differ within and between days (Amarger et al., 1979). Therefore, nitrogen fixation using ^15^N natural abundance is appropriate for estimating nitrogen fixation over the entire growing season and under different conditions (Sprent et al., 1996), especially when dealing with seeds. The measurement is based on the isotopic discrimination of δ^15^N isotope (Shearer and Kohl, 1986). Results showed that foliar B did not change the δ^15^N value in both watered and water stressed plants (Figures 3C,D). However, compared with watered plants (W–B and W+B), water stress resulted in higher δ^15^N value, indicating higher ^15^N. Carbon fixation source was investigated by measuring the change in δ^13^C. The values of δ^13^C in WS–B or WS+B were higher compared with W–B or B+B plants (the higher δ^13^C = less negative = less discriminated against).

## Discussion

### Effect of B on seed weight, nodule mass, and nitrogen fixation

The increase in seed weight and nodule mass indicated that foliar B had positive effects by increasing seed weight (100-seed weight and seed per plant), and may be due to its positive effects on nodule mass and higher nitrogen fixation rate. These results are in agreement with previous reports that B has indirect effects on nitrogen metabolism by increasing nitrogen demand (Shelp, 1993), stimulating nodule development, and increasing rate of nitrogen fixation capacity (Bolaños et al., 1994, 1996). Previous research also showed that there was little or no ability to fix N_2_ in B-deficient nodulated pea plants (Bolaños et al., 1994). Although there is no convincing evidence that B is directly involved in nitrogen metabolism, it was suggested that B protects nitrogenase from oxygen damage and maintains membrane integrity and function (Bonilla et al., 1997), and may interact with membrane polyhydroxy compounds such as glycoproteins and glycolipids to maintain the proper conformation in nitrogen-fixing cells (Brown et al., 2002). Our results suggested that foliar B increased seed weight, nodule mass, and nitrogen fixation rates. However, this positive effect of B on nitrogen fixation was shown in watered plants only (W+B) and not in water stressed plants (WS+B). In water stress plants no differences in nitrogen fixation rates were recorded between WS–B and WS+B (Figures 3A,B). The lower rate of nitrogen fixation in water stressed plants may be due to lower soil moisture and its effects on B uptake, nitrogen fixation, and plant growth (Frechilla et al., 2000). Previous research showed that nitrogen fixation is affected by environmental factors (Schubert, 1995), including water stress (Guerin et al., 1990; Pefia-Cabriales and Castellanos, 1993; Sinclair et al., 2007). Water stress affects nitrogen fixation by inhibiting *Rhizobium* (*Bradyrhizobium*) multiplication in soil, rhizobial infection of roots, nodulation, and N2 fixation rates (Schubert, 1995). It is well-established that nitrogen fixation is sensitive to drought (Sinclair and Serraj, 1995; Serraj et al., 1999; Frechilla et al., 2000). However, the interactions between atmospheric N required for nitrogen fixation and mineral nitrogen required for nitrogen assimilation need to be better understood in order to maximize N_2_ fixation and yield (George and Singleton, 1992).

### Effects of boron on seed protein, oil, and fatty acids

The higher seed protein concentration in watered plants supplied by foliar B could be due to the indirect effects of B on nitrogen fixation and protein synthesis. The higher seed protein concentration in water stressed plants compared with the control (W–B) could be due to the reduced seed weights, especially 100-seed weights, which indirectly reflect that seed size was lower under water stress. Same observation was recorded for oleic acid concentration where higher concentrations were recorded in W+B and WS–B and W+B (Table 2). Protein increase was accompanied by oil decrease, and oleic increase was accompanied by linoleic and linolenic acids decreases, supporting the genetic inheritance of the inverse relationship between protein and oil (Burton, 1985; Bellaloui et al., 2009b). Both palmitic and stearic fatty acids were relatively stable, supporting previous research (Bellaloui and Mengistu, 2008; Bellaloui et al., 2012a,b). Limited information is available on the effect of B on seed composition. However, the available previous research on B and seed composition showed a positive relationship between the level of B in soil and seed protein and oleic acid concentrations, and suggested that an indirect role of B in seed composition may exist (Bellaloui et al., 2009a). In addition, foliar B application resulted in higher soybean seed protein and oleic acid concentrations (Bellaloui et al., 2009b, 2010a). In spite of the inconsistent results on the effect of foliar fertilizers on seed composition, our results suggest that foliar B can alter seed composition, by increasing protein and oil and decreasing linoleic and linolenic acid.

The increase in oleic acid and decrease in linolenic acid by FB could be due to the effect of B on the activity of the enzymes, desaturases, involved in the accumulation and conversion of unsaturated fatty acids (oleic, linoleic, and linolenic acids). Since foliar boron was applied using water, the combined effects of both boron and water during the application may transiently impact photosynthesis and photo assimilate, especially for water stressed plants. Currently, there are no reports on how B is involved in the accumulation of seed oil or the conversion of unsaturated oleic and linolenic fatty acids. Further research is needed to better understand the mechanisms controlling this changes and environmental factors causing these changes.

### Effects of boron on seed sugars

The higher sucrose, glucose, and fructose levels with foliar B fertilization indicated B involvement in carbohydrate metabolism. Although the physiological roles of B involvement in sugar movement and metabolism were previous reported (Marschner, 1995; Brown et al., 1999; Perica et al., 2001), limited information on the role of B in seed sugars accumulation, especially under drought conditions. The decrease of sucrose, glucose, and fructose under water stress conditions indicated that these sugar types are sensitive to drought conditions, and applying foliar B can increase their levels in the seed. It appears that water stress can result in shift in sugars distribution in seed, maybe as an adaptive mechanism to drought stress. The influence of water stress on sugars was previous reported (Taji et al., 2002; Streeter, 2003; Bellaloui et al., 2012a,b). It was reported that raffinose and galactinol levels may play an important role in plant tolerance to biotic and abiotic stress (Bellaloui et al., 2012a,b), and the accumulation of galactinol and raffinose may protect the plant from stress environment, especially drought (Taji et al., 2002). Streeter (2003) and González et al. (1995) showed that the activity of sucrose synthase, the main enzyme involved in sucrose hydrolysis in nodules, was significantly inhibited under drought conditions. This indicated that sucrose, glucose, and fructose are more sensitive to water stress than raffinose and stachyose. I should be mentioned here that due to the high permeability of boron across membranes, foliar boron can enter the phloem and can complex sucrose, mobilizing B to the inflorescence, impacting sugar metabolism (Marschner, 1995; Bellaloui et al., 1999; Brown et al., 1999). So far, there is no clear evidence of the biological functions of raffinose and stachyose (Ren et al., 2009), but the oligosaccharides (sucrose, raffinose, and stachyose) are related to seed quality (Wilson, 2004) and the acquisition of desiccation tolerance during seed development and maturation, and protection of seeds against damage during seed dehydration and aging. The increase of sugars, especially raffinose and stachyose, may indicate the importance of these sugar fractions in protecting the plants under abiotic stress, in our case drought. It was reported that the accumulation of compatible solutes such as sugars may protect plants against environmental stress (Chen and Murata, 2002). Other researchers reported that non-structural carbohydrates (sucrose, hexoses, and sugar alcohols) were observed, and a strong correlation between sugar accumulation and osmotic stress tolerance was found (Streeter et al., 2001; Taji et al., 2002). It was suggested that the increase in sugars was a result of starch hydrolysis and sugars conversion (Ingram et al., 1997). Also, it was hypothesized that sugars act as osmotica and/or protect specific macromolecules and contribute to the stabilization of membrane structures, protect cells during desiccation (Phillips et al., 2002), and interact with polar headgroups of phospholipids in cell membranes to prevent membrane fusion. In our experiment, stachyose accumulation occurred under water stress, and this in agreement with other reports that many seeds accumulate considerable amounts of raffinoase oligosaccharides (RFOs) such as raffinose and stachyose. These sugars are thought to play a role in the acquisition of desiccation tolerance, and overexpression of galactinol synthase (catalyzes the first step in the biosynthesis of RFOs) resulted in higher accumulation of galactinol and raffinose and improved drought tolerance (Taji et al., 2002). The real mechanisms of how these compounds are involved in stress tolerance still not fully understood (Chen and Murata, 2002; Bartels and Sunkar, 2005), and further research is needed.

From the soybean industry perspective, soybean seed with high raffinose and stachyose concentrations are undesirable and have negative effects on the nutritive value of soymeal and are indigestible by humans and animals, especially monogastric animal such as chicken and pigs, causing flatulence or diarrhea (Liu, 1997). Low raffinose and stachyose levels in soybean seed are desirable (Obendorf et al., 1998) and high level of seed sucrose is desirable because it improves taste and flavor of tofu, soymilk, and natto (Hou et al., 2009). The relationship between sucrose, raffinose, and stachyose is not well-established and can be affected by genotype and environment, and their interactions. A positive correlation between total sugar and sucrose and raffinose was found, but no significant correlation was found for stachyose (Hartwig et al., 1997; Hou et al., 2009). A positive correlation was found between sucrose and raffinose, but a negative correlation was found between sucrose and stachyose (Hymowitz et al., 1972). Hou et al. (2009) evaluated 241 soybean plant introductions and concluded that the negative relationship between sucrose and oligosaccharides is desirable to increase sucrose content and reduce raffinose and stachyose. So far, no soybean cultivars with improved sugar profiles have been released (Hou et al., 2009), and further research to increase desirable sugars through breeding or agricultural practices is needed.

### Foliar boron effects on boron in leaves and seed

Our previous research showed that soybean grown in soil of 0.72 mg B kg^−1^ accumulated between 35 and 50 mg kg^−1^ in the fully expanded leaves, and this leaf B concentration represented adequate/sufficient B concentration for normal growth (Jones et al., 1991; Fageria et al., 1997; Parker and Pilbeam, 2007). Under water stress, B concentration in the fully expanded leaves ranged from 20 to 25 mg B kg-1, which is considered low level of B in soybean and inhibited growth, nodule mass and number, and nitrogen fixation (Bellaloui et al., 2010a; Bellaloui, 2011). The positive response of leaves and seed to B under water stress conditions may indicate that, even under adequate concentration of B in soil, soybean grown under drought conditions may require additional supply foliar B fertilizer to increase and maintain B levels in leaves and seed. Our experiment showed that a concentration of B <20 mg B kg^−1^ in the fully expanded leaves is a critical concentration at which foliar B can be applied for normal growth and high yield and seed quality. In The increased contribution of cell wall B to the total B, especially in water stressed plants (WS–B and WS+B), indicated the significant structural role of B (Hu and Brown, 1994; Hu et al., 1996), especially under drought conditions where B uptake is inhibited. Leaf water potential, resulted from water stress, could be a possibility to limit B uptake, and this is true, especially if we consider that B uptake is mostly a passive process and largely determined by the rate of water uptake through the plasma membrane of root cells (Hu and Brown, 1997) and the flow through water channels (Dordas et al., 2000), although active B uptake mechanism was also reported (Dannel et al., 2000, 2002). Since the main function role of B is structural and since cell wall boron constitutes about up to 71% (Bellaloui and Brown, 1998) or 95–98% (Matoh et al., 1992; Hu and Brown, 1994) under B-limiting conditions of cellular B, it was suggested that most of boron available in cell under B-deficiency condition is present in the cell wall. It was reported that the majority of this cell wall B (>70%) was associated with pectin (Matoh et al., 1993; Hu and Brown, 1994). Boron-pectin complexes in the cell walls determine B requirements by higher plants (Tanaka, 1967; Loomis and Durst, 1992). The possible explanation for the high percentage of cell B under water stress, shown in our experiment, is that plants under water stress suffered moderate B deficiencies, resulted from inhibition of B uptake due to water stress. Under these conditions, plants may sense through signals that B level in plant tissues is limited and what is available is used first for cell structure until the process of cell structure is satisfied. Any additional available B can be then used for metabolic functions.

### Effects of foliar boron and water stress on seed natural abundance of δ^15^N and δ^13^C isotopes

The higher δ^13^C values in seed of water stressed compared with watered plants indicated that a shift in δ^13^C isotope values and δ^13^C enrichment occurred. Foliar B did not result in any shifts or δ^13^C enrichment (Figures 3E,F). The non-shift in δ^15^N and δ^13^C by foliar B indicated that B does not affect the natural abundance of δ^15^N and δ^13^C. However, the enrichment of δ^15^N and δ^13^C in water stressed plants compared with watered plants indicated that water stress can change δ^15^N and δ^13^C values, leading to higher enrichment of δ^15^N and δ^13^C the higher δ^15^N, the higher ^15^N enrichment; the higher (less negative) δ^13^C, the higher ^13^C enrichment. The altered δ^15^N under water stress were indicated by increasing ^15^N (derived from soil nitrogen that is used for nitrate assimilation) and decreasing ^14^N (derived from atmospheric nitrogen that is used for nitrogen fixation). The possible mechanisms of how plants shift δ^15^N and compensate for the inhibition of nitrogen fixation under water stress to minimize the damage and maintain nitrogen uptake, movement, and protein synthesis are not well-understood. However, the observation of a higher δ^15^N under water stress indicated that plants may use soil nitrogen as a source of nitrogen to compensate for the sensitivity and inhibition of nitrogen fixation, and this could be a possible physiological mechanism because nitrogen fixation is more sensitive to water stress than nitrogen assimilation. It has been reported that the δ^15^N values in the xylem and plant tissues is associated with the acquired N, and the value can be altered because of N metabolism (Yoneyama et al., 1997; Bellaloui and Mengistu, 2008; Bellaloui et al., 2008).

The increase in δ^13^C or higher ^13^C/^12^C ratio (less negative) in seed under water stress conditions indicated that drought altered the source of carbon fixation. It was reported that the δ^13^C value in plant tissues can be influenced by water supply and temperature (O'Leary, 1995), plant physiology (Kumarasinghe et al., 1992), and mycorrhizal infection (Högberg, 1990). The abundance of δ^13^C in plant tissues may reflect the effects of environmental conditions on plant gas exchange as associated with stomatal conductance and CO_2_ fixation (Livingston et al., 1999). For example, drought stress can lead to stomatal closure and ^13^C fixation increase, resulting in less discrimination against δ^13^C (Sun et al., 1996; Matsushima, 2007). The shift in ^13^C/^12^C ratio indicates a shift in carbon fixation metabolism from ribulose bisphosphate (RuBP) carboxylase pathway to phosphoenolpyruvate carboxylase (PEP), resulting in δ^13^C enrichment (O'Leary, 1995). During carbon fixation by photosynthesis, the naturally occurring stable isotope ^13^C is discriminated against, and plants would have a smaller ^13^C to ^12^C ratio than ^13^C to ^12^C ratio in fixed CO_2_ of the air, suggesting a possible use of this technique to select for water use efficiency (Farquhar et al., 1989). Our results demonstrated that δ^15^N and δ^13^C values changed and enrichment occurred under water stress conditions, suggesting that both nitrogen and carbon metabolism pathways were affected, impacting seed protein, oil, and sugar synthesis.

## Conclusion

The current research demonstrated that foliar B application increased seed protein, oleic acids, glucose, and fructose concentrations under watered/irrigated conditions. The increase of protein was accompanied by decrease in oil, and the increase of oleic acid was accompanied by a decrease of linoleic and linolenic acids, reflecting the inverse relationships between these constituents. The increase of seed protein, oleic acid, and stachyose under water stress condition may be due to water stress effects and seed weight decrease, reflecting the potential roles of oleic acid and stachyose in plant tolerance to biotic and abiotic stresses. Foliar B application resulted in B increase in both leaves and seed under watered and water stressed conditions, and foliar B application may be beneficial under water stress conditions. It must be noticed here that normally, under optimal conditions, the plants may not respond positively to FB because of continuous adequate supply of B. However, in our case, although watered plants had adequate B in the soil, FB resulted in an increase in nitrogen fixation and some seed composition components. This response can be explained as follows: under midsouth conditions and during the growing season of soybean, temperature during reproductive stages, especially seed-fill stage can reach up to more than 39°C in the field or greenhouse conditions, and this temperature may have created suboptimal conditions due to the heat effect, impacting B uptake and translocation. Under these conditions, plants may positively respond to foliar applied boron. This could be the reason why under sufficient B and under well-watered conditions FB B had a stimulatory effect on nitrogen fixation and some seed composition components.

The high percentage of cell wall B to the total B, especially under water stress conditions, reflects the structural role of B. The change of ^14^N values reflects use of soil nitrogen, indicating a possible mechanism switch by the plant to use nitrogen assimilation rather than nitrogen fixation to compensate for the loss of nitrogen fixation capacity under water stress. The change of carbon isotope may be correlated with water use efficiency, and under watered conditions the naturally occurring stable isotope ^13^C is discriminated against during carbon fixation and plants would have a smaller ^13^C to ^12^C ratio than ^13^C to ^12^C ratio in fixed CO2 of the air (Farquhar and Richards, 1984). However, under water stress/drought and due to stomatal closure and lower conductance, ^13^C fixation increase, leading to less ^13^C discrimination (Fotelli et al., 2003; Hanba et al., 2003). Our research demonstrated that water stress/drought can alter ^15^N and ^13^C, probably by altering nitrogen and carbon fixation pathways.

### Conflict of interest statement

The authors declare that the research was conducted in the absence of any commercial or financial relationships that could be construed as a potential conflict of interest.
